# Cyanobacterial biomass as carbohydrate and nutrient feedstock for bioethanol production by yeast fermentation

**DOI:** 10.1186/1754-6834-7-64

**Published:** 2014-04-17

**Authors:** K Benedikt Möllers, David Cannella, Henning Jørgensen, Niels-Ulrik Frigaard

**Affiliations:** 1 Department of Biology, University of Copenhagen, Strandpromenaden 5, 3000 Helsingør, Denmark; 2 Department of Geosciences and Natural Resource Management, University of Copenhagen, Rolighedsvej 23, 1958 Frederiksberg C, Denmark; 3 Department of Chemical and Biochemical Engineering, Technical University of Denmark, Søltofts Plads, Building 229, 2800 Kgs Lyngby, Denmark

**Keywords:** Cyanobacteria, Bioethanol, Microalgae, *Saccharomyces*, Yeast extract

## Abstract

**Background:**

Microbial bioconversion of photosynthetic biomass is a promising approach to the generation of biofuels and other bioproducts. However, rapid, high-yield, and simple processes are essential for successful applications. Here, biomass from the rapidly growing photosynthetic marine cyanobacterium *Synechococcus* sp. PCC 7002 was fermented using yeast into bioethanol.

**Results:**

The cyanobacterium accumulated a total carbohydrate content of about 60% of cell dry weight when cultivated under nitrate limitation. The cyanobacterial cells were harvested by centrifugation and subjected to enzymatic hydrolysis using lysozyme and two alpha-glucanases. This enzymatic hydrolysate was fermented into ethanol by *Saccharomyces cerevisiae* without further treatment. All enzyme treatments and fermentations were carried out in the residual growth medium of the cyanobacteria with the only modification being that pH was adjusted to the optimal value. The highest ethanol yield and concentration obtained was 0.27 g ethanol per g cell dry weight and 30 g ethanol L^-1^, respectively. About 90% of the glucose in the biomass was converted to ethanol. The cyanobacterial hydrolysate was rapidly fermented (up to 20 g ethanol L^-1^ day^-1^) even in the absence of any other nutrient additions to the fermentation medium.

**Conclusions:**

Cyanobacterial biomass was hydrolyzed using a simple enzymatic treatment and fermented into ethanol more rapidly and to higher concentrations than previously reported for similar approaches using cyanobacteria or microalgae. Importantly, as well as fermentable carbohydrates, the cyanobacterial hydrolysate contained additional nutrients that promoted fermentation. This hydrolysate is therefore a promising substitute for the relatively expensive nutrient additives (such as yeast extract) commonly used for *Saccharomyces* fermentations.

## Background

Photosynthetic biomass is a promising resource for the generation of biofuels and other valuable bioproducts. However, rapid biomass production and high-yield conversion processes are essential for successful applications. Plant-derived lignocellulosic biomass is abundant but, due to the recalcitrant nature of this material, significant challenges have to be solved if this biomass is to be used for the microbial production of biofuels and bioproducts [1-3]. Photosynthetic microorganisms constitute an appealing alternative source of biomass for many reasons. Photosynthetic microorganisms grow much faster than terrestrial plants, have a higher efficiency in using the energy of light, and can be cultivated in areas and in a manner that do not compete with plant-based food and feed production [4-7]. In this context, marine photosynthetic microorganisms have a distinct advantage in large-scale cultivation as they can be cultivated in sea water, which is not suitable for human consumption and most agricultural uses.

The most abundant photosynthetic microorganisms in nature are cyanobacteria and certain eukaryotic microalgae, including green algae, red algae, and diatoms [8]. Despite their plant-like photosynthesis, the evolutionary origins and cellular properties of these microorganisms are very diverse. Cyanobacteria produce a wealth of high-value bioproducts and have been mass-cultivated for centuries as a nutritional supplement [9]. Currently, much effort is being put into the genetic and metabolic engineering of photosynthetic microorganisms, especially cyanobacteria, for the production of bioproducts not naturally produced by these organisms [10,11]. However, the direct use of biomass from cyanobacteria and other microalgae as a feedstock for the generation of biofuels and other bioproducts is underexplored [12,13].

In terms of biomass utilization, cyanobacteria have certain advantages over eukaryotic microalgae. In spite of the overall Gram-negative-like structure of the cell envelope, the cyanobacterial cell wall contains a peptidoglycan layer that more closely resembles that of Gram-positive bacteria [14]. The cell wall in cyanobacteria is therefore degradable by lysozyme, and is less complex and less diverse than the cell walls of most microalgae, which consist of a wide range of complex polysaccharides and proteoglycans [15]. Furthermore, the type of storage carbohydrate is of vital importance if the biomass is to be used as a fermentation substrate for fungi. Cyanobacteria have glycogen as a storage carbohydrate [16,17]. Glycogen is not found in any eukaryotic microalgae, which typically have either starch (green algae, red algae) or β-glucans (brown algae, diatoms) as the main storage carbohydrate [18]. Both glycogen and starch are essentially α-1,4-glucans with α-1,6-branching but glycogen particles are small (0.04–0.05 μm) and water-soluble, whereas starch particles are much larger (0.1–100 μm) and water-insoluble [18]. Thus, glycogen may be preferred over starch as a fermentation feedstock because *in vitro* starch mobilization by heating and enzymatic treatment is a more energy-intensive process than glycogen mobilization [3].

Green microalgae and cyanobacteria typically accumulate starch or glycogen to a content of 10 to 50% of their biomass, depending on the strain and growth conditions, and this polysaccharide is potentially useful as substrate for biofuel fermentation [12]. Whole-cell material from starch-enriched green microalgae [19-22] and glycogen-enriched cyanobacteria [13] has recently been used as feedstock for bioethanol production by yeast fermentation. These studies employed various enzymatic, chemical, and physical treatments (including drying, heating, and acid- and base-treatment) to liberate monomeric hexoses from the biomass. In the present work, the single-celled, marine cyanobacterium *Synechococcus* sp. PCC 7002 (hereafter denoted *Synechococcus*; previously known as *Agmenellum quadruplicatum* PR-6; [23]) was used as a biomass feedstock for anaerobic fermentation by the yeast *Saccharomyces cerevisiae*. This *Synechococcus* strain accumulates glycogen and cyanophycin as carbon and nitrogen storage compounds and does not produce polyhydroxybutyrate as is observed in some cyanobacteria [24-26]. Exhaustion of nitrate in the growth medium of *Synechococcus* causes well-coordinated and complex physiological adaptations that allow photosynthesis and growth to continue to some extent [27-30]. This results in an increased C:N ratio of the biomass, an increased carbohydrate content (mostly glycogen), and a degradation of nitrogenous components including the light-harvesting phycobilisome (PBS) antenna proteins [24,30].

The objectives of the present work were to investigate if whole-cell, carbohydrate-loaded *Synechococcus* biomass treated only with enzymes is suitable as fermentation feedstock, and to explore the ethanol productivity when using a high concentration of this biomass as fermentation feedstock. We show that *Synechococcus* biomass indeed could be sufficiently degraded by enzymatic treatment, and that it served both as fermentable substrate and as nutrient source for the fermenting yeast. This resulted in higher ethanol productivity than previously reported with whole-cell biomass from microalgae [19-22] or cyanobacteria [13]. In addition, the present study also suggests that enzymatically hydrolyzed cell material from cyanobacteria could have a general use as a nutrient supplement to enhance the yeast fermentations of various biomass feedstocks low in nitrogenous compounds and other nutrients.

## Results and discussion

### Nitrate limitation and carbohydrate accumulation in *Synechococcus*

**Figure 1:**
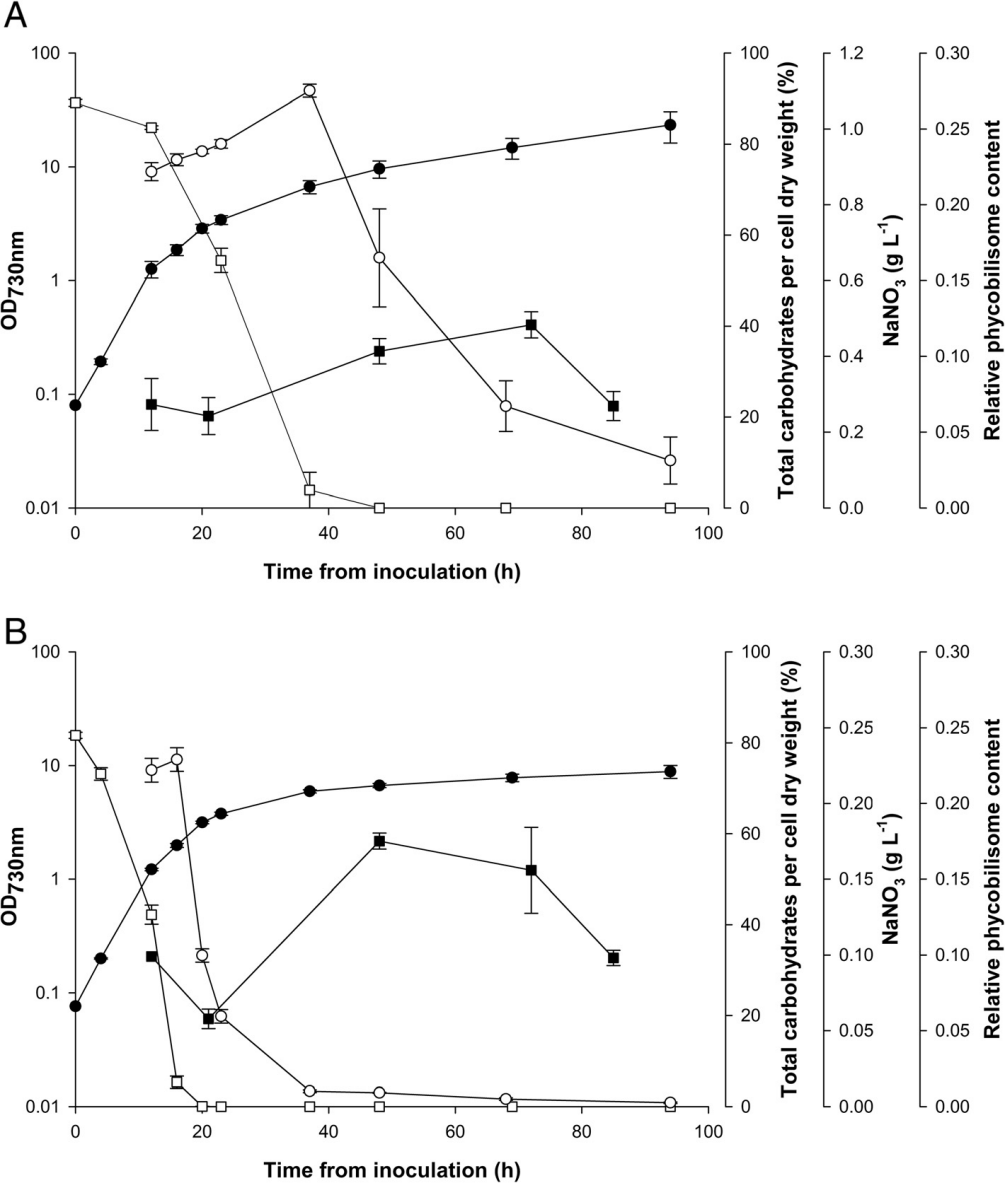
**Cultivation of *Synechococcus *with different nitrate concentrations. (A)** Initial NaNO_3_ concentration of 1 g L^-1^. **(B)** Initial NaNO_3_ concentration of 0.24 g L^-1^. Solid circles: OD_730_ (optical density at 730 nm); solid squares: total carbohydrates per cell dry weight (% weight per weight); open squares: NaNO_3_ concentration (g L^-1^); open circles: relative phycobilisome content per cell. h, hours.

*Synechococcus* was cultivated in medium A supplemented with nitrate to various concentrations (0.12 to 1.0 g NaNO_3_ L^-1^) to determine how the total carbohydrate content and other cellular parameters varied over time (Figure 1, Table 1, and in Additional file 1: Figures S1 and S2). In cell cultures with nitrate present, the total carbohydrate content per dry weight (DW) was roughly between 20 and 35% weight per weight (w/w) depending on growth conditions (Figure 1 and in Additional file 1: Figure S2C). Upon nitrate depletion, growth slowed and the total carbohydrate content increased dramatically (Figure 1). The maximum total carbohydrate accumulation depended on the initial nitrate concentration and the cell density at the time of nitrate depletion. The highest total carbohydrate content per DW was about 60% w/w determined in cultures with initially 0.24 or 0.36 g NaNO_3_ L^-1^ (Figure 1B, Table 1, and in Additional file 1: Figure S2). A higher initial nitrate concentration of 1 g NaNO_3_ L^-1^ resulted in a higher cell density but also a lower maximum total carbohydrate content per DW (about 40% w/w; Table 1). Thus, inoculation of *Synechococcus* to an optical density at 730 nm (OD_730_) of about 0.1 into a medium with 0.24 g NaNO_3_ L^-1^ and a cell harvest 48 hours after inoculation was used to obtain nitrogen-depleted biomass with maximum carbohydrate accumulation (about 60% w/w; Figure 1B and Table 1).

**Table 1:** **Maximum total carbohydrate content obtained in *Synechococcus *** **cell cultures with various nitrate concentrations**^**1**^

**NaNO**_**3 **_**concentration (g L**^**-1**^**)**	**Time from inoculation (hours)**	**Cell DW (g L**^**-1**^**)**	**Total carbohydrates per cell DW (%)**
0.12	21	0.90 ± 0.15	52 ± 8
0.24	48	1.65 ± 0.05	58 ± 2
0.36	72	3.0 ± 0.2	59 ± 4
1	72	3.7 ± 0.3	40 ± 3

^1^Determined from time course experiments (in Additional file 1: Figure S2). DW, dry weight.

**Figure 2:**
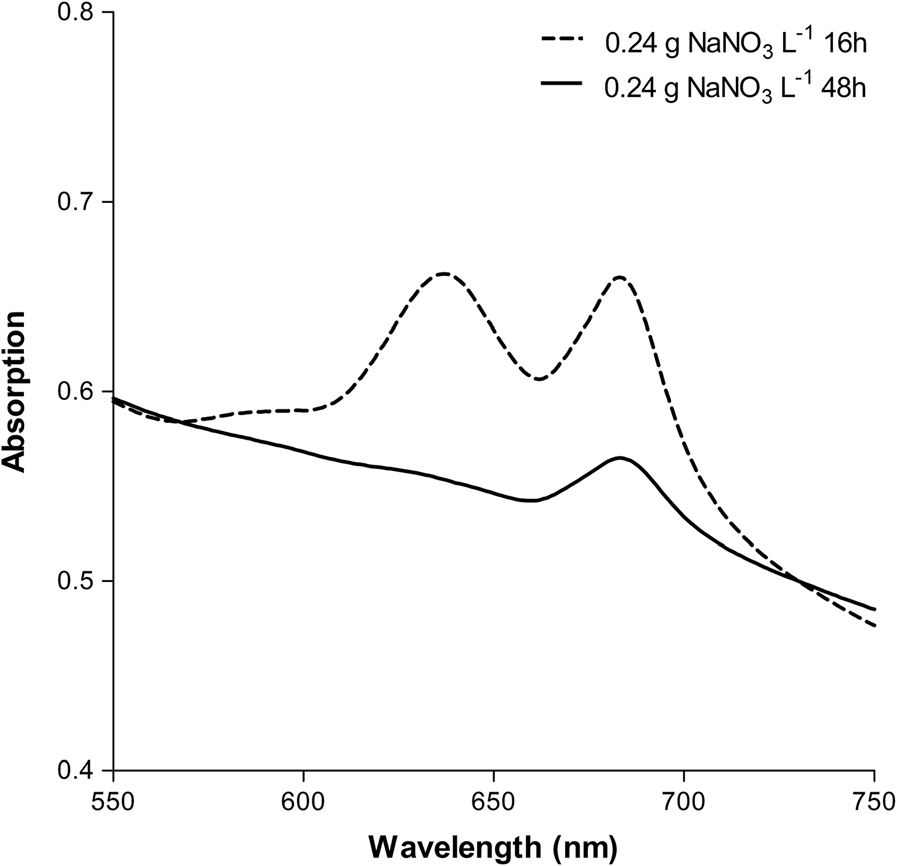
**Absorption spectra of intact *Synechococcus *cells.** Nitrate-replete cells (at 16 hours with 0.24 g NaNO_3_ L^-1^; dashed line) and nitrate-limited cells (at 48 hours with 0.24 g NaNO_3_ L^-1^; solid line). The spectra were recorded in dilute cell suspensions (OD_730_ = 0.3 to 0.4) and normalized to an OD_730_ of 0.5. The peak at 637 nm is due to phycobilisomes and the peak at 683 nm is due to chlorophyll *a*. OD_730_, optical density at 730 nm.

Upon prolonged incubation after nitrate depletion, the total carbohydrate content decreased (Figure 1 and in Additional file 1: Figure S2C). This may be caused by physiological stress in the cells that grew poorly due to nitrogen limitation, which eventually caused consumption of the storage carbohydrates probably for maintenance of cellular functions. Thus, for preparation of cells with maximum carbohydrate accumulation, the harvest was performed prior to this stage where the relative content of carbohydrate decreased. In this context, it is interesting to note that immediately following nitrate depletion, simultaneous degradation of PBS and chlorophyll (Chl) *a* commenced (Figures 1 and 2 and in Additional file 1: Figure S1). In the culture with 0.24 g NaNO_3_ L^-1^, PBS was over 95% degraded within 24 hours after nitrate depletion; the total carbohydrate content peaked within another 10 to 15 hours after PBS was almost completely degraded (Figure 1B). Labeling experiments in *Arthrospira platensis* have shown that most of the glycogen synthesized upon nitrate depletion is derived from intracellular protein, predominantly from PBS [31], and the same is likely to be the case in *Synechococcus*. In addition, the capacity for glycogen synthesis has been shown to correlate with the capacity for the degradation of pigments (especially PBS) in nitrate-limited cultures of cyanobacterial strains deficient in glycogen synthesis [32-35]. This may explain why the maximum in carbohydrate accumulation apparently coincided with complete, or nearly complete, degradation of PBS. Thus, the progress of PBS degradation in *Synechococcus* is a useful proxy for estimating both the initiation of nitrate limitation and the timing of maximum carbohydrate accumulation. From a process control perspective, the advantage is that PBS can be easily and rapidly monitored, possibly online, by measuring the PBS absorbance of culture samples.

In order to produce sufficient amounts of biomass for fermentation trials, the culture volumes were scaled up to 800 mL. A comparison of growth (as OD_730_) and other key cellular parameters in 25 mL and 800 mL culture setups showed little difference (Additional file 1: Figure S3).

### Biochemical composition of *Synechococcus*

**Figure 3:**
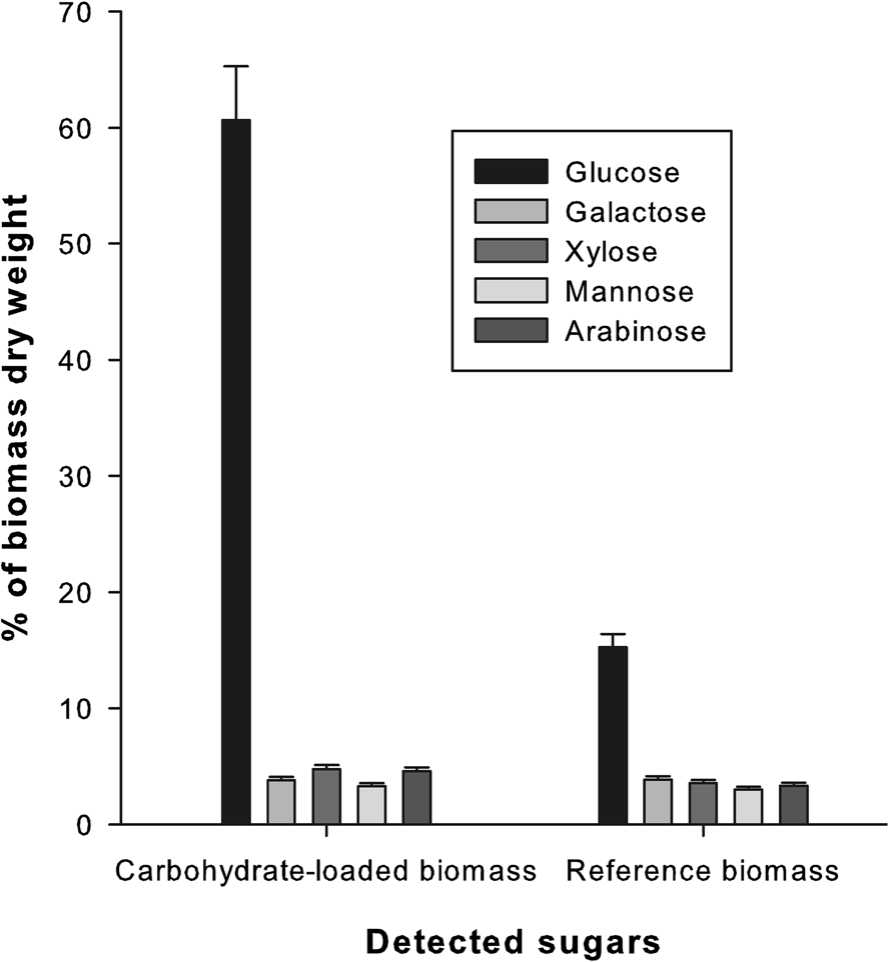
**Monosaccharide analysis of *Synechococcus *biomass.** Cells were cultivated for 48 hours with either low (0.24 g NaNO_3_ L^-1^) or high (1.0 g NaNO_3_ L^-1^) initial nitrate concentrations to generate ‘carbohydrate-loaded biomass’ or ‘reference biomass’, respectively.

The monosaccharide composition of acid-hydrolyzed *Synechococcus* cells grown for 48 hours with 0.24 g or 1 g NaNO_3_ L^-1^ is shown in Figure 3. Clearly, the only monosaccharide that increased dramatically during nitrate-limited conditions was glucose, which accounted for about 60% w/w of DW in the nitrogen-depleted biomass. Most of this increase was due to accumulation of glycogen [32,35,24,36]. The content of other monosaccharides measured was rather similar in the two samples tested, in the manner of 3 to 5% w/w of DW (Figure 3).

The carbon content of *Synechococcus* cell DW was 49 ± 2% w/w (*n* = 2) for cultures that were nitrate replete and 47 ± 0% w/w (*n* = 2) for cultures that were nitrate limited, which is very similar to values previously obtained with cyanobacteria [37]. Previously obtained C:N weight ratios with the same *Synechococcus* strain were 5.8 during growth with nitrate (similar to the Redfield Ratio of 5.7 generally observed in marine microalgae that are not nutrient-limited [38]), 3.4 during growth with ammonium, and up to 13 during nitrogen starvation (assuming a carbon content of 48% w/w of DW) [30]. In our cultures, exponentially growing *Synechococcus* cells in the presence of nitrate (25 hours with 1 g NaNO_3_ L^-1^; Figure 1) had a C:N weight ratio of 4.4 ± 0.4 (*n* = 2). The *Synechococcus* cells with maximum carbohydrate accumulation obtained from cultivation with 0.24 g NaNO_3_ L^-1^ for 48 hours (60% w/w total carbohydrate; Figure 1) had a C:N weight ratio of 11 ± 1.4 (*n* = 2). A prolonged incubation of these *Synechococcus* cultures for 120 hours (0.24 g NaNO_3_ L^-1^; Figure 1A) resulted in a slightly increased C:N weight ratio of 12.2 ± 1.3 (*n* = 2), even though the total carbohydrate content dropped to 35% w/w. Thus, a high C:N ratio is not necessarily indicative of a high carbohydrate content. Presumably the carbohydrate was eventually eliminated by respiration and other metabolic processes.

### Enzymatic hydrolysis of *Synechococcus*

**Figure 4:**
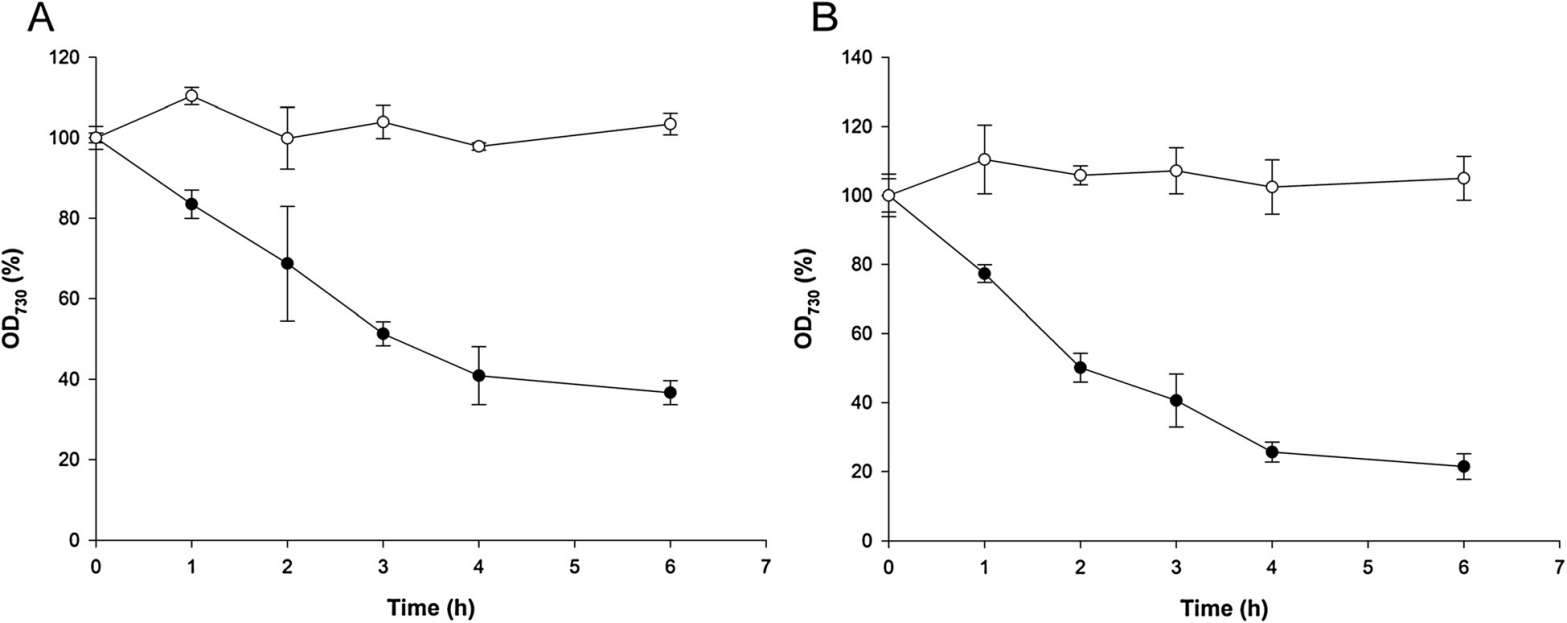
**Effect of lysozyme on *Synechococcus *cell disintegration.** Cells were cultivated for 48 hours with either low (0.24 g NaNO_3_ L^-1^) or high (1.0 g NaNO_3_ L^-1^) initial nitrate concentrations to generate ‘carbohydrate-loaded biomass’ or ‘reference biomass’, respectively. Cell suspensions were concentrated five times (by centrifugation and re-suspension in a smaller volume of the supernatant) and stored at either at 4°C (open circles) or at -20°C (solid circles) for 1 hour prior to incubation with lysozyme (100 mg L^-1^) at 37°C. Cell disintegration was followed over time by measuring the OD_730_ after appropriate dilution of small aliquots of the reaction mixture into water. The OD_730_ is depicted as percentage of the OD_730_ at the start of the lysozyme treatment. **(A)** Reference *Synechococcus* cells. **(B)** Carbohydrate-loaded *Synechococcus* cells. h, hours; OD_730_, optical density at 730 nm.

Efficient release of carbohydrates from cells by disintegration of the cellular structure is important for the exploitation of cyanobacteria as a production platform for fermentation. Lysozyme is an antibacterial enzyme that hydrolyzes the glycosidic linkages in the peptidoglycan of bacterial cell walls, and is therefore a potentially useful enzyme for the disintegration of bacterial cells [39]. The disintegration of *Synechococcus* cells by lysozyme was studied by measuring the OD_730_ of cell suspensions (Figure 4). OD_730_ is a suitable measure of cell integrity because the debris from lysed cells is too small to cause scattering of light at 730 nm [40]. When freshly harvested cells were incubated at 4°C in the residual growth medium, subsequent lysozyme treatment at 37°C had little effect on the cells. However, if freshly harvested cells were frozen at -20°C for 1 hour in the residual growth medium and then treated with lysozyme at 37°C, cells clearly disintegrated as observed by a decrease in OD_730_. Investigation by microscopy showed that cells in these freeze-treated and lysozyme-treated suspensions changed morphology from regular sized and ovoid-shaped cells to swollen, spherical cells due to osmotic effects and eventually appeared to disintegrate. This behavior is typical of cyanobacteria and other bacteria when the cell wall is degraded by lysozyme in a hypotonic solution [39,41]. *Synechococcus* cells that were only freeze-treated and not exposed to lysozyme did not change morphology and did not lyse. Thus, freeze treatment of *Synechococcus* cells appeared to destabilize the cell structure and increase the susceptibility to lysozyme. Cells grown with high and low nitrate concentrations behaved similarly with respect to disintegration by lysozyme (Figure 4).

**Figure 5:**
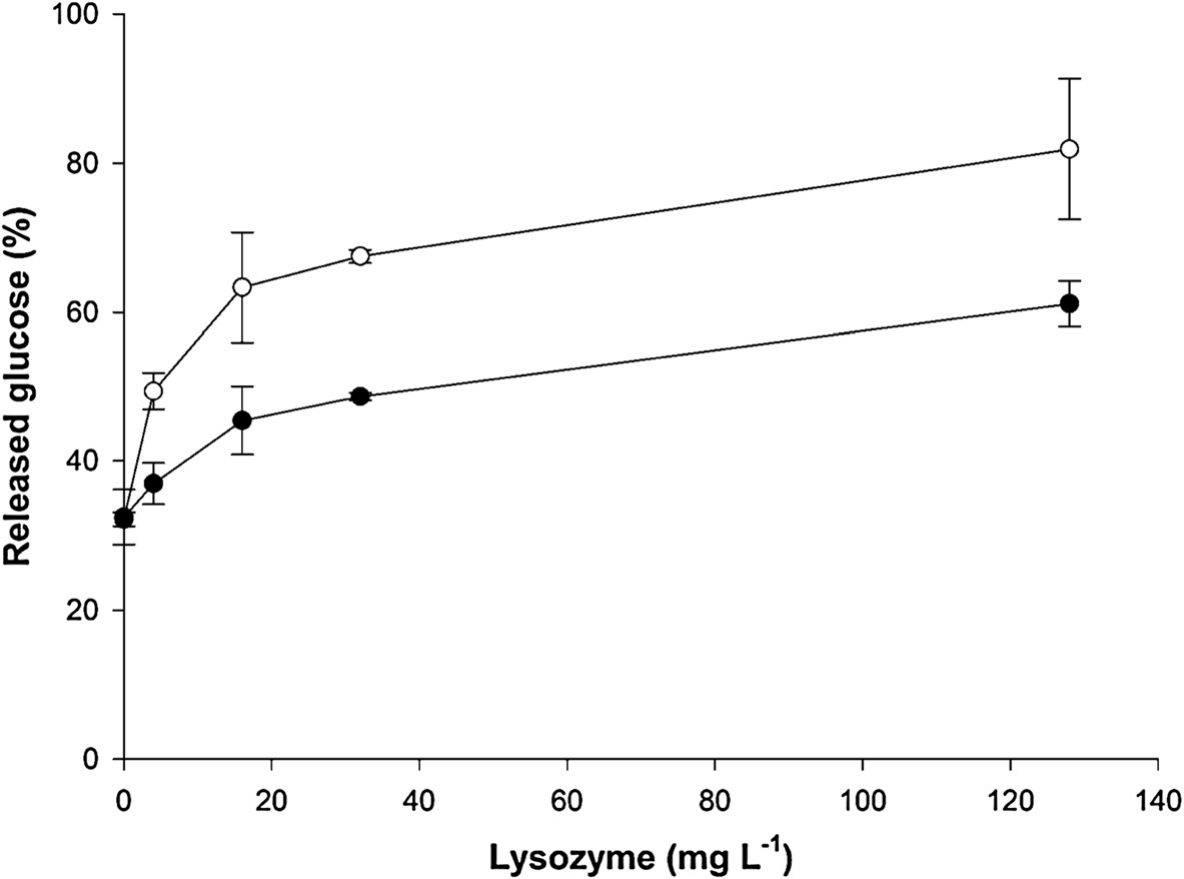
**Effect of lysozyme on glucose mobilization from *Synechococcus *hydrolysate.** Carbohydrate-loaded *Synechococcus* biomass (see Figure 3) were hydrolyzed by treatment with lysozyme and alpha-glucanases as described in the methods and materials section (including incubation at 34°C for 48 hours after addition of Spirizyme® Fuel), except that the freshly-harvested cell paste were either stored at 4°C (solid circles) or at -20°C (open circles) for 1 hour prior to enzyme treatments and that the lysozyme concentration was varied as indicated. The released glucose in the supernatant of the biomass-enzyme mixture was determined by high-performance liquid chromatography. The total glucose concentration from the added biomass was about 15 g L^-1^ (as determined by monosaccharide analysis of acid-hydrolyzed biomass) and corresponds to 100% in the figure.

Lysis of *Synechococcus* cells and subsequent mobilization of intracellular carbohydrates were obtained using the combined treatment of lysozyme and two alpha-glucanases (Liquozyme® SC DS and Spirizyme® Fuel). The alpha-glucanases are used for the industrial processing of starch-containing materials and were used here according to the manufacturer’s recommendation (see Methods). Therefore, the additional effect of lysozyme on the glucose mobilization from *Synechococcus* biomass was studied (Figure 5). The results showed that freeze-treating the cells and increasing the concentration of lysozyme promoted release of glucose from the *Synechococcus* biomass. Interestingly, even without sonication or lysozyme treatment, applying the two alpha-glucanases liberated a significant amount of glucose from the cells: ~30% of the total glucose in the biomass (as determined by monosaccharide analysis of acid-hydrolyzed biomass) and this glucose mobilization was not dependent on whether the cells had been frozen (Figure 5). The origin of this glucose is not clear since the cells did not disintegrate in the absence of lysozyme. A previous report suggests that unknown glucans, possibly including cell-surface-associated exopolysaccharides, accumulate under certain conditions in *Synechococcus* (such as nitrate limitation) even when glycogen biosynthesis has been genetically eliminated, and that glucose may be liberated from these unknown glucans by externally added alpha-glucanases [35]. Thus, a proportion of the total amount of glucose that can be mobilized from *Synechococcus* biomass may originate from cellular structures other than intracellular glycogen.

In conclusion, to promote disintegration of *Synechococcus* cells and mobilization of monomeric glucose for fermentation by *S. cerevisiae*, the cells were treated with freezing, lysozyme, and two alpha-glucanases.

### Fermentation of *Synechococcus* hydrolysate

**Figure 6:**
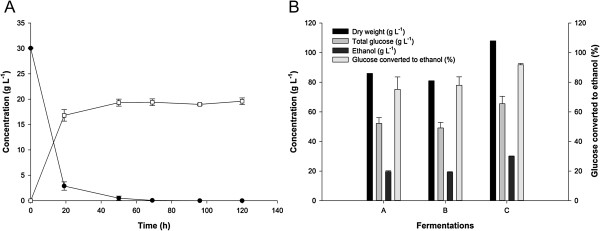
**Ethanol fermentation of *Synechococcus *hydrolysate by *S. cerevisiae*. (A)** Fermentation time course of *Synechococcus* hydrolysate in medium A. Open squares: ethanol; solid circles: glucose. **(B)** Fermentation end results of three different batches of *Synechococcus* hydrolysates (time course of fermentation C is shown in panel A). h, hours.

Hydrolysates produced from carbohydrate-loaded *Synechococcus* cells were fermented using an industrial strain of *S. cerevisiae*. Figure 6A shows a representative time course of a fermentation experiment in which the *Synechococcus* hydrolysate is used as sole carbon and nutrient source. In the depicted experiment, the *Synechococcus* hydrolysate with an initial DW content of 86 g L^-1^ yielded 17 g ethanol L^-1^ after 20 hours, and 19 g ethanol L^-1^ after 48 hours, equivalent to a final yield of 0.22 g ethanol per g DW or 0.37 g ethanol per g glucose. The free glucose concentration at the onset of fermentation was 30 g L^-1^_,_ which corresponds to about 60% of the total glucose in the biomass (as determined by monosaccharide analysis of acid-hydrolyzed biomass). Thus, not all glucose was released from the *Synechococcus* biomass at the onset of fermentation. The enzymatic hydrolysis of the polysaccharides continued after inoculation of the yeast, as is the case for the simultaneous saccharification and fermentation (SSF) approach applied in the starch-to-ethanol industry [1,3]. Glycerol was detected (3 to 4 g L^-1^) as the major byproduct in the fermentation of *Synechococcus* hydrolysate [42]. Acetate and lactate were detected but at concentrations less than 1 g L^-1^. The highest ethanol concentration obtained by fermentation of a *Synechococcus* hydrolysate was 30 g L^-1^ from a hydrolysate with 108 g DW L^-1^ equivalent to a yield of 0.27 g ethanol per g DW (Figure 6B, fermentation C). The conversion of glucose to ethanol in this fermentation corresponded to more than 90% of the theoretical maximum (0.51 g ethanol per g glucose). These experiments demonstrate that the *Synechococcus* biomass can be readily fermented, even at high biomass loadings, and thereby that the biomass does not contain compounds inhibitory to the yeast.

**Figure 7:**
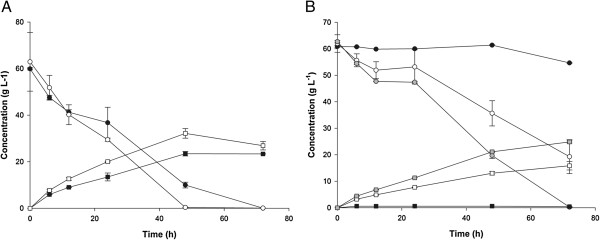
**Ethanol fermentation of glucose solutions by *S. cerevisiae*. (A)** Fermentation time course of glucose (60 g L^-1^) in medium A with (open symbols) and without (solid symbols) added *Synechococcus* hydrolysate (1.0 g DW hydrolysate L^-1^). Squares: ethanol; circles: glucose. **(B)** Fermentation time course of glucose (60 g L^-1^) in water without (solid symbols black) and with added *Synechococcus* hydrolysate (open symbols: 1.0 g DW hydrolysate L^-1^; closed symbols grey: 4.3 g DW hydrolysate L^-1^). Squares: ethanol; circles: glucose. h, hours.

Besides acting as a carbon source during fermentation, lysis of the *Synechococcus* cells could release other compounds, which could serve as nutrients for the yeast during the fermentation. Previously it has been shown that nutrients and especially complex organic nitrogen sources can improve fermentation performance in high gravity brewing/fuel ethanol production [43,44] or fermentation of lignocellulosic hydrolysates [45]. To study this, we tested the effect of adding *Synechococcus* hydrolysate to yeast fermentations of pure glucose solutions. Inclusion of even small amounts of *Synechococcus* hydrolysate (1.0 to 4.3 g DW L^-1^) in a solution of glucose (60 g L^-1^) in either medium A (Figure 7A) or water (Figure 7B) significantly increased the glucose consumption and ethanol production rate. In medium A, the ethanol concentration was 50% higher after 24 hours with *Synechococcus* hydrolysate supplementation than without (Figure 7A). The highest rates of ethanol production within the initial 24 hour of fermentation (15 to 20 g ethanol L^-1^ day^-1^) were obtained with *Synechococcus* hydrolysate in medium A and with glucose in medium A supplemented with *Synechococcus* hydrolysate. Addition of a complex organic nutrient source, typically yeast extract or corn steep liquor, is commonly found to be beneficial for *Saccharomyces* fermentations of nitrogen-poor substrates [45,46]. The results presented here confirm that *Synechococcus* hydrolysate has a similar beneficial effect on fermentation of glucose by *S. cerevisiae*. The components in the hydrolysate responsible for this effect are not known but could include vitamins and other nitrogenous compounds such as amino acids. Recent findings with nitrate-limited cyanobacteria suggest that, due to active protein hydrolysis, increased amounts of proteases and free amino acids could be available in the hydrolysate [31], which potentially could result in improved yeast performance.

To this day, very few studies have investigated the fermentation of whole-cell material from cyanobacteria and microalgae. In a recent study intact cells of the cyanobacterium *A. platensis* were added to a concentration of about 20 g cell DW L^-1^ to a fermentation medium containing lysozyme (1 g L^-1^), yeast extract (10 g L^-1^), and peptone (20 g L^-1^) [13]. The fermenting organism was a recombinant amylase-producing strain of *S. cerevisiae*, which eliminated the need for externally added alpha-glucanases. A final ethanol concentration of 6.5 g L^-1^ was obtained. The fermentation rate was low (1.08 g ethanol L^-1^ day^-1^), but a high ethanol yield was obtained (0.325 g ethanol per g cell DW). Similar to our study, the authors observed that lysozyme greatly enhanced the ethanol yield and that the available glucose was efficiently converted to ethanol (86% of theoretical maximum). The higher yield and rates obtained in our work are probably due to the fact that the cyanobacterial cells were partially degraded by enzymatic pretreatment prior to inoculation of yeast, thus making the glucose readily available for the yeast. An important observation from our work is that additional nutrients to support the fermenting yeast such as yeast extract and/or peptone were not necessary in our approach.

From an industrial point of view, a high final ethanol concentration is critical as it lowers energy consumption and thereby the costs of distillation. Typically 40 to 50 g ethanol L^-1^ is regarded as the lower threshold for economic ethanol production [1], but final ethanol concentrations exceeding 100 g ethanol L^-1^ are common in the fuel ethanol industry [47]. Two important factors limit high final ethanol concentrations from the fermentation of biomass obtained from cyanobacteria and microalgae: the DW content in the fermentation and the carbohydrate content of the DW. If the biomass of cyanobacteria and microalgae obtained from liquid cultures is not dried, the DW content of the wet biomass obtained by centrifugation or filtration is typically not more than 10 to 20% w/w [13]. In our case we managed to obtain a maximum carbohydrate accumulation of 60% of DW. Given these limitations, an expected maximum yield is approximately 60 g ethanol L^-1^, which is well above the critical ethanol concentration for distillation. However, efficient and cost effective harvest and dewatering of the biomass remain to be the most critical process steps.

## Conclusions

In this work, carbohydrate-enriched biomass from cyanobacteria was obtained by photoautotrophic cultivation under nitrate limitation. This biomass was converted to a substrate suitable for fermentation by *S. cerevisiae* by simple enzymatic pretreatment. Harsh chemical treatments or separation of carbohydrate-containing fractions of the biomass were not necessary to obtain a high final ethanol concentration (30 g L^-1^). The maximum carbohydrate accumulation in the cyanobacterial biomass coincided with the degradation of PBS, which implies that PBS may be used as a proxy for carbohydrate accumulation in cyanobacteria in general. Although the cyanobacterial biomass was successfully used for bioethanol production alone, this biomass may be more valuable as a carbohydrate and/or nutrient source for production of products more valuable than ethanol in a biorefinery concept. This may be to supply nutrients and additional sugar to the fermentation of lignocellulosic materials low in nitrogen and other nutrients.

## Methods

### Cultivation of *Synechococcus* and *Saccharomyces*

*Synechococcus* sp. PCC 7002 (referred to as *Synechococcus*) was a kind gift from Donald A. Bryant (The Pennsylvania State University, PA, USA). It was grown in medium A supplemented with 1 g NaNO_3_ L^-1^[48] unless otherwise stated. Liquid cultures were bubbled with a constant flow of air supplemented with 1% v/v CO_2_ provided by a gas mixer (GMS150, Photon Systems Instruments, Drasov, Czech Republic). Small-scale cultures (25 mL) were maintained in tubes with an inner diameter of 2.2 cm and large-scale cultures (800 mL) were maintained in bottles with an inner diameter of 9.5 cm. All culture vessels were placed in the middle of a 30-liter transparent tank with thermostatically controlled water at 38°C. Constant illumination was provided by fluorescent tubes (cool white light; Philips Master TL-D, 18 W/840; Philips Electronics, Amsterdam, The Netherlands) placed against two opposite sides of the tank. Small-scale cultures were illuminated by 250 μmol photons s^-1^ m^-2^. Large-scale cultures were illuminated by 250 μmol photons s^-1^ m^-2^ at the time of inoculation. After 12 hours of growth (OD_730_ ≈ 1) the irradiance of large-scale cultures was increased to 400 μmol photons s^-1^ m^-2^. Small-scale cultures were inoculated to an OD_730_ of 0.2 to 0.3 and large-scale cultures were inoculated to an OD_730_ of approximately 0.1. In growth experiments where the effect of nitrate was studied, *Synechococcus* cells were harvested by centrifugation at ambient temperature (5000 g for 2 minutes), re-suspended in medium A, and used as inoculum. Reported measurements on *Synechococcus* growth experiments are the average and standard deviation of biological duplicates (Figures 1 and 3, Table 1, and in Additional file 1: Figures S1, S2 and S3). The *S. cerevisiae* strain Thermosacc® Dry (Lallemand Inc., Montreal, Canada) was used to ferment *Synechococcus* hydrolysates. The yeast was aerobically pre-cultured in CBS medium [49] for 48 hours prior to each fermentation experiment. For preparation of *Saccharomyces* cells for inoculation of fermentation reactions, 100 mL of fresh *Saccharomyces* culture was harvested by centrifugation at 4100 rpm for 10 minutes and re-suspended in 10 mL of 0.9% NaCl solution in water. The cell DW of the yeast suspension was measured and adjusted to 50 g L^-1^ prior inoculation of the hydrolysates.

### Hydrolysis and fermentation of *Synechococcus* biomass

*Synechococcus* cultures to be used for fermentation were harvested by centrifugation for 20 minutes at 10.000 g (50 mL Falcon tubes) and cell pellets were stored at -20°C until use. Cell pellets were thawed and re-suspended to a slurry by the addition of a small volume of medium A using mild sonication (Misonix S-4000; Qsonica, Newtown, Connecticut, United States) with a cup horn (amplitude 50%, 2 minutes processing time, 5 second on/off cycle) to obtain a final DW content of about 100 g L^-1^ (pH approximately 7). The subsequent enzyme treatments of the biomass were carried out in the cyanobacterial growth medium without the addition of buffers or other chemical agents. Lysozyme (from chicken egg white, L6876, Sigma-Aldrich, St. Louis, United States) was added (100 mg L^-1^) and the solution was incubated for 3 hours at 37°C. Two alpha-glucanases (Liquozyme® SC DS and Spirizyme® Fuel; Novozymes A/S, Bagsværd, Denmark) were used according to the instructions by the manufacturer. Liquozyme® SC DS (240 α-amylase units per g) was added (0.21% w/w) and the mixture incubated at 85°C for 1.5 hours. Then another aliquot of Liquozyme® SC DS was added (0.14% w/w) and the mixture incubated at 60°C for 0.5 hours. The pH was then adjusted to 5.5–6.0 with 5 M HCl. Finally Spirizyme® Fuel (750 amyloglucosidase units per g) was added (0.08% w/w). This mixture, now denoted ‘hydrolysate’, was then used for fermentation without further treatment. Experiments with varying lysozyme concentrations were carried out in duplicates (Figures 4 and 5).

Hydrolysates (3 mL) were inoculated with freshly concentrated yeast suspension (60 μL) to a final yeast cell concentration of 1 g DW L^-1^. The fermentations were run in 10 mL glass vials with a pressure resistant lock. The headspace was flushed with nitrogen gas prior to incubation in an orbital shaker (160 rpm) oven at 34°C. Fermentation experiments with pure *Synechococcus* hydrolysates were carried out in biological triplicates (Figure 6). Fermentation experiments with glucose solutions supplemented with *Synechococcus* hydrolysates were carried out in biological duplicates (Figure 7).

### Optical measurements

The optical density of *Synechococcus* cultures at 730 nm (OD_730_) was used as a measure of cell density [40]. The content of PBS and Chl *a* per cell in arbitrary units was estimated from their absorption maxima corrected for optical scattering as follows:

$$\begin{array}{l} \text{PBS}\;\text{content}=\left[{\text{OD}}_{637}-{\text{OD}}_{730}-0.55\left({\text{OD}}_{560}-{\text{OD}}_{730}\right)\right]/{\text{OD}}_{730}\\ \text{Chl}\;a\;\text{content}=\left[{\text{OD}}_{683}-{\text{OD}}_{730}-0.28\left({\text{OD}}_{560}-{\text{OD}}_{730}\right)\right]/{\text{OD}}_{730} \end{array}$$

Cell suspensions were diluted to an OD_730_ between 0.1 and 0.6 prior to measurement. All measurements were performed with a UV1800 spectrophotometer (Shimadzu, Kyoto, Japan).

### Dry weight determination and chemical analyses

The cell DW in *Synechococcus* cultures was determined by separating the cells from the liquid by filtration (Whatman GF/F filters, GE Healthcare, Little Chalfont, United Kingdom). The filters were pre-dried for 24 hours at 90°C, then loaded with 1 ml of culture (0.5 mL after 48 hours of growth) and oven dried for 24 to 48 hours at 90°C. The DW content in hydrolysates, yeast cultures, and biomass suspensions was determined by drying the material on aluminum pans inserted in a thermogravimetric moisture analyzer (Sartorius, Göttingen, Germany). Carbon and nitrogen elemental analysis was performed on dried biomass using a Flash 2000 NC Soil Analyzer (Thermo Scientific, Waltham, Massachusetts, United States). Nitrate was determined in the supernatant of cell cultures after removal of the cells by centrifugation using a cadmium-reduction-based nitrate test kit (cat. no. 3319, LaMotte, Maryland, United States). Total carbohydrate was determined using the phenol sulfuric acid assay [50].

The monosaccharide composition of *Synechococcus* biomass was analyzed by strong acid hydrolysis using the TAPPI (Technical Association of the Pulp and Paper Industry) standard procedure [42], except that the standard curve samples were treated identically to the biomass samples to correct for sugar degradation [51]. The monosaccharides D-glucose, D-xylose, L-arabinose, D-mannose, and D-galactose were measured on a ICS5000 system (Dionex, Sunnyvale, California, United States) equipped with a CarboPac PA1 2 × 50 mm guard column and 2 × 250 mm separating column (Dionex), operated at a flow of 0.25 mL min^-1^ and maintained at 30°C. Prior to detection, a post column flow of 0.1 mL min^-1^ of 0.2 M NaOH solution was mixed together with the flow carrying the separated sugars and analyzed using a PAD gold detector (Dionex).

The fermentation broth was analyzed for glucose, mannose, lactate, acetate, glycerol, and ethanol using an Ultimate 3000 HPLC (Dionex, Germering, Germany) equipped with an RI-101 refractive index detector (Shodex, Yokohama, Japan) and UV detector at 210 nm (Dionex). The separation was performed on a Rezex ROA column (Phenomenex, Torrance, California, United States) at 80°C with 5 mM H_2_SO_4_ as eluent at a flow rate of 0.6 mL min^-1^.

Dry weight determinations and chemical analyses were carried out in technical duplicates.

## Abbreviations

Chl *a*: chlorophyll *a*; DW: dry weight; OD: optical density; PBS: phycobilisome; w/w: weight per weight.

## Competing interests

The authors declare that they have no competing interests.

## Authors’ contributions

KBM: conception and design, data collection and analysis, manuscript writing and final approval of the manuscript. DC: conception and design, data collection and analysis, critical revision and final approval of the manuscript. HJ: conception and design, data analysis, financial support, manuscript writing, final approval of manuscript. NUF: conception and design, data analysis, financial support, manuscript writing, final approval of manuscript. All authors read and approved the final manuscript.

## Supplementary Material

Additional file 1 **Supplemental material on cultivation experiments with *Synechococcus *** **referred to within the manuscript.** Click here for file
