# Supplementary material for: Cyanobacterial biomass as carbohydrate and nutrient feedstock for bioethanol production by yeast fermentation
Source: Biotechnol Biofuels. 2014 Apr 17;7:64. doi: 10.1186/1754-6834-7-64 (PMC4022056; doi:10.1186/1754-6834-7-64)
Supplement: Additional file 1 — Supplemental material on cultivation experiments with Synechococcus referred to within the manuscript. [file 1754-6834-7-64-S1.pdf]

# Supplemental material

K. B. Möllers, D. Cannella, H. Jørgensen, and N.-U. Frigaard

*Biotechnology for Biofuels* 2014

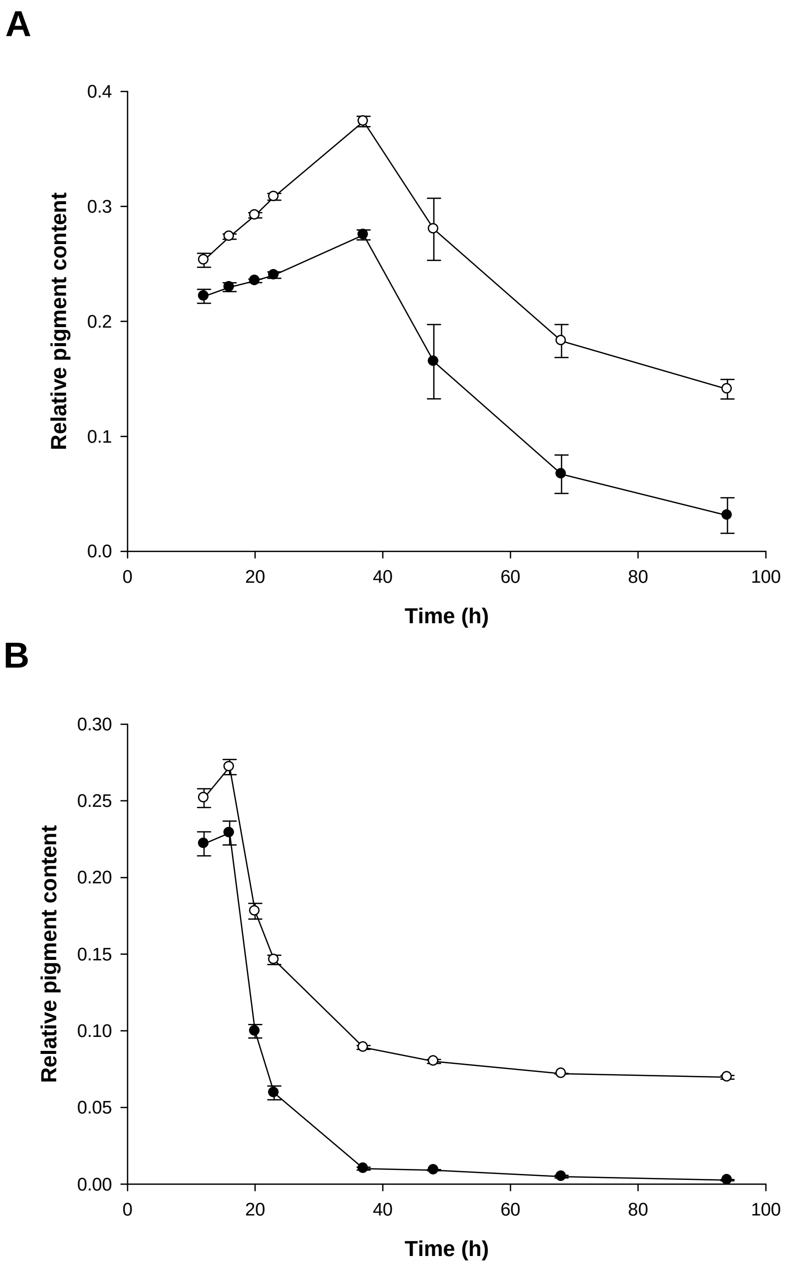


**Figure S1. Relative pigment content in *Synechococcus* cultures with different initial nitrate concentrations.** (**A**) Initial $NaNO_3$ concentration of 1.0 g $L^{-1}$. (**B**) Initial $NaNO_3$ concentration of 0.24 g $L^{-1}$. (See Fig. 1 for nitrate concentrations.) Open circles: relative chlorophyll *a* content per cell; closed circles: relative phycobilisome (PBS) content per cell. Values represent the average and standard deviation of two biological replicates.

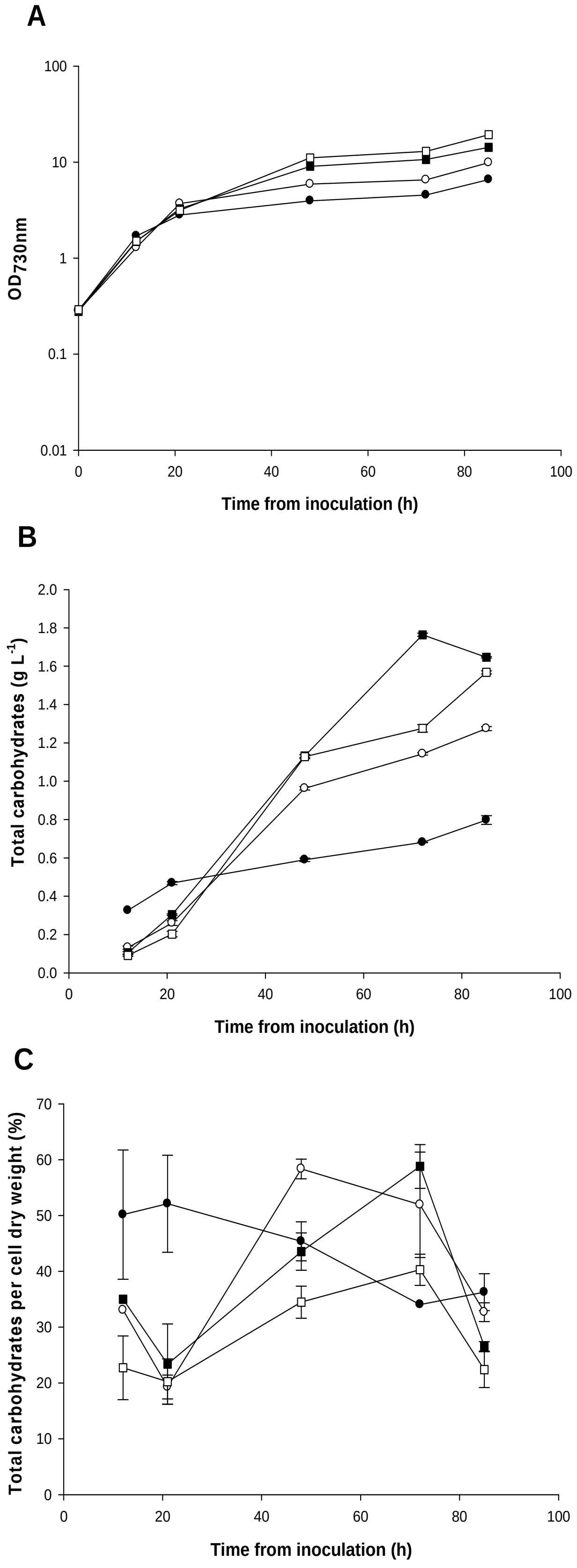


**Figure S2. Growth of *Synechococcus* in medium A supplemented with different $NaNO_3$ concentrations.** (**A**) $OD_{730}$. (**B**) Total carbohydrate accumulation. (**C**) Total carbohydrates per cell dry weight (% w/w). Closed circles: initial $NaNO_3$ concentration of 0.12 g $L^{-1}$; open circles: initial $NaNO_3$ concentration of 0.24 g $L^{-1}$; closed squares: initial $NaNO_3$ concentration of 0.36 g $L^{-1}$; open squares: initial $NaNO_3$ concentration of 1 g $L^{-1}$. Values represent the average and standard deviation of two biological replicates.

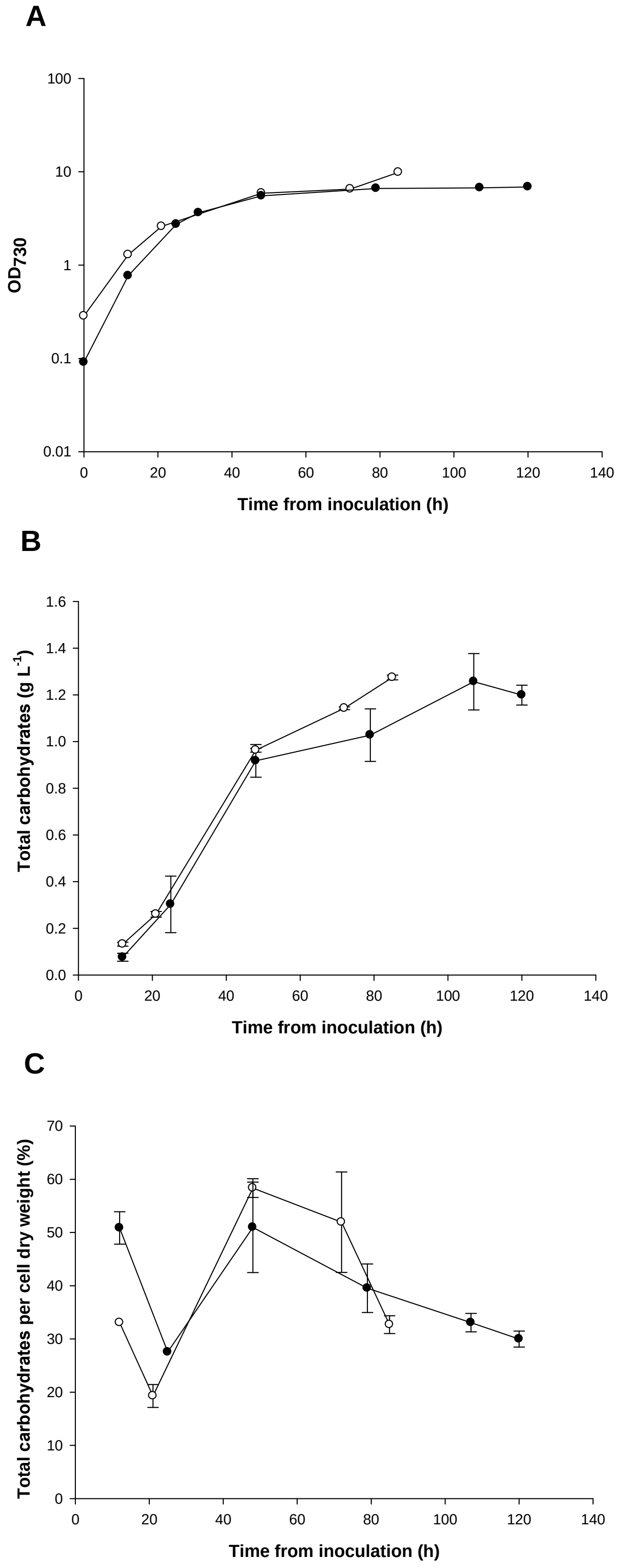


**Figure S3. Growth of *Synechococcus* in 25-mL and 800-mL cultures in medium containing 1 g $NaNO_3$ $L^{-1}$.** (**A**) $OD_{730}$. (**B**) Total carbohydrate accumulation. (**C**) Total carbohydrates per cell dry weight (% w/w). Open circles: 25 mL cultures; closed circles: 800 mL cultures. Values represent the average and standard deviation of two biological replicates.
